# A phase II, multicenter, single-arm trial of eribulin as first-line chemotherapy for HER2-negative locally advanced or metastatic breast cancer

**DOI:** 10.1186/s40064-016-1833-1

**Published:** 2016-02-24

**Authors:** Tsutomu Takashima, Shinya Tokunaga, Seika Tei, Shigehiko Nishimura, Hidemi Kawajiri, Shinichiro Kashiwagi, Shigehito Yamagata, Satoru Noda, Takeo Nishimori, Yoko Mizuyama, Takeshi Sunami, Kenji Tezuka, Katsumi Ikeda, Yoshinari Ogawa, Naoyoshi Onoda, Tetsuro Ishikawa, Shinzoh Kudoh, Minoru Takada, Kosei Hirakawa

**Affiliations:** Department of Surgical Oncology, Osaka City University Graduate School of Medicine, 1-4-3 Asahimachi Abeno, Osaka, 5458585 Japan; Osaka City General Hospital, Osaka, Japan; Seichokai Fuchu Hospital, Izumi, Japan; Sumitomo Hospital, Osaka, Japan; Ishikiri Seiki Hospital, Higashi-Osaka, Japan; Ikuwakai Memorial Hospital, Osaka, Japan; Ohno Memorial Hospital, Osaka, Japan; Izumi Municipal Hospital, Izumi, Japan; Kinki-Cho Chest Medical Center, Sakai, Japan; Kashiwara Municipal Hospital, Kashiwara, Japan; Osaka Socio-Medical Center Hospital, Osaka, Japan; Hanwa Daini Senboku Hospital, Sakai, Japan

**Keywords:** Metastatic breast cancer, HER2-negative breast cancer, Eribulin mesylate, Objective response rate, Survival

## Abstract

The treatment goals for metastatic breast cancer (MBC) are prolonging survival and improving the quality of life. Eribulin, a non-taxane tubulin inhibitor, demonstrated improved survival in previous studies and also showed mild toxicity when used in late-line therapy for MBC. We conducted a phase II study to investigate the efficacy of eribulin mesylate as the first-line chemotherapy for human epidermal growth factor receptor 2 (HER2)-negative MBC. This was a phase II, open-label, single-arm, multicenter trial conducted in Japan. Patients with HER2-negative MBC received intravenous eribulin (1.4 mg/m^2^ on days 1 and 8 of each 21-day cycle). The primary efficacy outcome was overall response rate (ORR). Secondary outcomes included time to treatment failure, progression-free survival (PFS), overall survival (OS), and safety. A total of 35 patients were enrolled and received a median of 8 (range 1–21) cycles of eribulin therapy. ORR and clinical benefit rate were 54.3 and 62.9 %, respectively. Median PFS was 5.8 months and median OS was 35.9 months. Grade 3 or 4 neutropenia was observed in 63 % of patients. The majority of non-hematological adverse events were mild in severity. The present trial demonstrated that eribulin has antitumor activity comparable with other key established cytotoxic agents with acceptable safety and tolerability. Thus, eribulin as first-line chemotherapy might be beneficial for patients with HER2-negative MBC.

## Background

The prognosis for patients with human epidermal growth factor receptor 2 (HER2)-positive metastatic breast cancer (MBC) has improved significantly since anti-HER2 therapies became commercially available. However, the long-term survival of patients with HER2-negative breast cancer remains poor, with a 5-year survival rate of only 24.3 % for distant metastatic disease (Howlader et al. 2013). As MBC is currently incurable, the goals of therapy are to prolong survival, palliate symptoms, and optimize quality of life (QoL) (Partridge et al. 2014). Anthracycline- or taxane-based regimens have often been chosen as first-line therapy for HER2-negative MBC. The current guidelines suggest using a single agent to optimize both treatment length and QoL for first-line therapy, except in the case of immediately life-threatening disease (Partridge et al. 2014; Cardoso et al. 2014). Based on these guidelines, agents with reduced toxicity but comparable efficacy to anthracyclines and taxanes could be therapeutic options for first-line therapy in such patients. In fact, a recent clinical study conducted in Japan demonstrated non-inferiority of the oral 5-fluorouracil derivative S-1 in overall survival (OS) and superiority in QoL against taxane as first-line chemotherapy for MBC (Takashima et al. 2016).

Recently, eribulin, a non-taxane microtubule dynamics inhibitor belonging to the halichondrin class of antineoplastic agents, which has a mechanism of action distinct from currently available taxanes (Jordan et al. 2005; Smith et al. 2010), has become available for treatment of MBC. In a phase 3, open-label, randomized trial (EMBRACE study), eribulin showed a significant and clinically meaningful improvement in OS compared to treatment of the physician’s choice in patients with heavily pretreated MBC (Cortes et al. 2011). In a different trial, the survival benefit of eribulin was similar to that of capecitabine in patients with MBC who had previously been treated with anthracycline- and taxane-based regimens (Kaufman et al. 2015). Moreover, the pooled analysis of those two trials demonstrated that eribulin significantly prolonged the OS compared with controls (Twelves et al. 2014). In addition to OS benefit, the non-hematological toxicity reported with eribulin treatment is mostly mild. These two findings suggest that eribulin would be a suitable option for early-line treatment of MBC to minimize toxicity and maximize survival benefit.

Although eribulin has been approved in Japan for the treatment of patients with inoperable or recurrent breast cancer, and is not limited to those who have been previously treated with chemotherapy regimens, data on first-line use of eribulin for treatment of Japanese patients with MBC are still limited. To date, only one phase II trial conducted outside Japan has included a small number of Asian patients with MBC (McIntyre et al. 2014). Moreover, current guidelines do not specify a preferred regimen for HER2-negative MBC. Therefore, we conducted a phase II trial to investigate the efficacy and safety of eribulin for first-line treatment of Japanese patients with HER2-negative MBC.

## Patients and methods

### Patients

Key inclusion criteria included: female patients with histologically confirmed HER2-negative MBC (including patients with unresectable advanced disease); aged ≥20 and <75 years; no history of chemotherapy for MBC other than peri-operative therapy (patients who received hormone therapy, immunotherapy, or local radiotherapy for MBC could be included in this trial); at least 6 months since the last administration of neoadjuvant or adjuvant chemotherapy; Eastern Cooperative Oncology Group performance status (ECOG PS) of 0 or 1; having measurable lesion(s) based on the Response Evaluation Criteria in Solid Tumors (RECIST) ver. 1.1 (New response evaluation criteria in solid tumours 2009); and adequate bone marrow, liver, renal, and lung functions. Key exclusion criteria included: hypersensitivity to eribulin; systemic infection; uncontrolled pleural effusion/ascites or pericardial effusion; symptomatic brain tumor; serious complications, active concomitant malignancy; pregnancy (including possible pregnancy) of premenopausal women. Patients who were considered ineligible by the investigator were also excluded.

### Study design

This was a phase II, open-label, single-arm, multicenter trial conducted at eight sites in Japan. The study protocol and all amendments were approved by local ethics committees or the institutional review board at each study site. This trial was conducted in accordance with the Japanese Guidelines for Clinical Research of the Ministry of Health, Labor and Welfare and the Declaration of Helsinki, as well as other applicable regulatory requirements. All participants provided written informed consent prior to study entry. The present trial has been registered with the University Hospital Medical Information Network (UMIN) Center (ID: UMIN000006086). This was an investigator-initiated clinical trial that was not supported by any industry funding, nor requested by any organization.

Eribulin was administered intravenously, without any premedication, at a dose of 1.4 mg/m^2^ over 2–5 min on days 1 and 8 of a 21-day cycle (2-weeks-on, 1-week-off). For patients who were not eligible for administration of eribulin on day 8 (i.e., neutrophil count <1000/mm^3^, platelet count <75,000/mm^3^, ≤grade 2 non-hematological adverse events), the next cycle started on day 22. The dose was reduced to 1.1 mg/m^2^ if one of the following had occurred during the previous cycle: neutrophil count <500/mm^3^ for more than 7 days; presence of febrile neutropenia; grade 4 thrombocytopenia; and grade 3 or higher non-hematological toxicity. The dose was further reduced to 0.7 mg/m^2^ if there was a toxicity as described above despite dose reduction to 1.1 mg/m^2^. Patients who were refractory to eribulin were able to continue treatment based on the choice of the investigator. Concomitant use of other anticancer therapy (e.g., hormone therapy, targeted therapy, immune therapy, and chemotherapy other than eribulin) and any local therapy was prohibited. Concomitant use of bone modifying agents was permitted if the agents had been used since prior to the study entry. Use of granulocyte colony-stimulating factor was permitted, but not for prophylactic administration, by decision of the investigator based on the clinical practice guideline (Smith et al. 2006).

The primary efficacy outcome was overall response rate (ORR), defined as the proportion of patients who achieved a complete response (CR) plus those who achieved a partial response (PR). The secondary endpoints included progression-free survival (PFS), OS, time to treatment failure (TTF), and safety. Time to response and duration of response were also assessed.

### Assessment

The information on patients’ characteristics at baseline was collected within 28 days prior to the initiation of eribulin administration. Baseline tumor assessments by radiographic evaluation (e.g., computerized tomography or magnetic resonance imaging scans) were also performed within 28 days prior to the initiation of eribulin administration, and tumor assessments were performed by the same methods every 2 cycles thereafter. Tumor assessments were analyzed based on the RECIST ver. 1.1 and classified as CR, PR, stable disease (SD), progressive disease, or not evaluable. Tumor response was confirmed at least 4 weeks after the criteria for response were met. PFS was defined as the time from initiation of eribulin to disease progression or death from any cause, OS was defined as the time from initiation of eribulin to death from any cause, and TTF was defined as the time from initiation of eribulin to treatment discontinuation for any reason (e.g., disease progression, treatment toxicity, patient preference, or death). Time to response was the time from initiation of eribulin to documentation of tumor response and duration of response was defined as the time from documentation of tumor response to disease progression, which was assessed among patients who reached ORR. For safety, adverse events, physical examination, vital signs, laboratory tests, and tumor markers (i.e., carcinoembryonic antigen and breast cancer antigen 15-3) were assessed during the study. All adverse events were graded according to the Common Terminology Criteria for Adverse Events ver. 4.0 (CTEP 2015).

### Statistical analysis

The following assumptions were made to determine target enrollment. In the EMBRACE study (Cortes et al. 2011), ORR for patients who received eribulin after a median of four previously administered regimens was 12 %. In addition, ORR of nanoparticle albumin-bound paclitaxel and paclitaxel for MBC was reported as 33 and 19 %, respectively, in all patients and 42 and 27 %, respectively, in the subgroup (40 % of the full cohort) who received those agents as first-line therapy in the phase III trial (Gradishar et al. 2005). Based on those results, we set threshold and expected values of ORR as 20 and 40 %, respectively. To meet the threshold and expected values of ORR with 80 % power and one-sided alpha error of 0.05, at least 32 patients were needed. Thus, we aimed to enroll 35 patients with the expectation of approximately 10 % ineligible patients.

Primary efficacy outcome (proportion of patients who achieved CR or PR for at least 4 weeks) was assessed in the full analysis set, which included all patients who received at least one dose of eribulin. In addition, clinical benefit rate (CBR) was defined as the proportion of patients who achieved CR, PR, or SD for at least 24 weeks. The median values with 95 % confidence interval (CI) for PFS, and OS curves were estimated with the Kaplan–Meier method. TTF, time to response, and duration of response were presented as median values with ranges. The safety analysis was also conducted in the full analysis set. All statistical analyses were one-sided, and probability values of <0.05 were considered to indicate a statistically significant difference.

## Results

### Patients

A total of 35 patients with HER2-negative MBC were enrolled between September 2011 and May 2014; none were excluded from our primary analysis. The characteristics of the patients at baseline are summarized in Table 1. The median age was 64 years (range 40–75), and the all patients had ECOG PS 0 or 1. Twenty-eight patients (80 %) were hormonal receptor-positive. Ten patients (29 %) received perioperative chemotherapy with anthracycline and/or taxane and five patients (14 %) received perioperative chemotherapy with other agents. The median number of cycles of eribulin administration was 8 (range 1–21), and the median relative dose intensity per week was 91.6 % (range 44.7–100 %). Dose modification was needed in four patients, and schedule modification in 19 patients. Patients were followed up for a median of 23.0 months (range 1.0–48.6) at data cut-off (October 15, 2015).

**Table 1:** Patient characteristics at baseline

Variable	n
Patients	35
Median age years (range)	64 (40–75)
Menopause	
Pre	9
Post	26
ECOG PS	
0	28
1	7
Hormone receptor	
Positive	28
Negative	7
Stage	
Inoperable	14
Recurrent	21
Neoadjuvant/adjuvant chemotherapy	
Yes	15
No	20
Prior anthracycline	10
Prior taxanes	9
No. of metastatic sites	
1	17
2	12
3	5
4	1
Metastatic site	
Lung	17
Bone	12
Liver	7
Lymph node	12
Pleura	3
Skin	2
Adrenal	1
Treatment exposure, cycles (range)	8 (1–21)
Dose reduction	
Yes	4
No	31
Schedule modification	
Yes	19
No	16

*ECOG PS,* Eastern Cooperative Oncology Group performance status

### Efficacy analysis

**Fig. 1:**
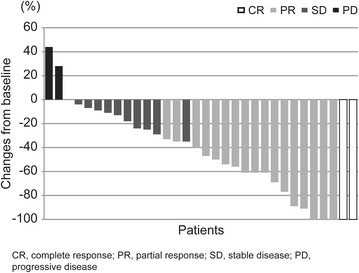
Percentage change in total sum of target lesion diameters from baseline to postbaseline nadir

The ORR was 54.3 % (95 % CI 37.8–70.8) and CBR was 62.9 % (95 % CI 46.8–78.9) (Table 2; Fig. 1). Among eight patients with locally advanced disease, four discontinued eribulin therapy and were able to undergo surgery as a result of down-staging. In the subgroups stratified by estrogen receptor status of the tumor, ORR for patients with luminal-like disease and those with triple-negative disease was somewhat similar; 53.6 % (95 % CI 35.1–72.0) and 57.1 % (95 % CI 20.5–93.8), respectively. ORR for patients with a disease-free interval of <2 or ≥2 years was similar. On the other hand, ORR for patients who did not receive any neoadjuvant/adjuvant chemotherapy was higher at 70.0 % (95 % CI 49.9–90.1) compared to 33.3 % (95 % CI 9.5–57.2) in those who received neo/adjuvant chemotherapy. In addition, ORR for patients who received neo/adjuvant chemotherapy without anthracycline- or taxane-based regimens was higher at 64.0 % (95 % CI 45.2–82.8) compared to 30.0 % (95 % CI 1.6–58.4) in those who received anthracycline- or taxane-based neo/adjuvant chemotherapy. Moreover, patients without visceral metastasis had higher ORR at 66.7 % (95 % CI 40.0–93.3) compared to 47.8 % (95 % CI 27.4–68.2) in those with visceral metastasis.

**Table 2:** Overall response rate

n (%)	Overall	Neoadjuvant/adjuvant chemotherapy				Subtype		Visceral metastasis		Disease free interval		
		Yes	No	With A/T	W/O A/T	Luminal	TN	Yes	No	<2 yr	≥2 yr	W/O Op.
Patients	35 (100)	15 (42.9)	20 (57.1)	10 (28.6)	25 (71.4)	28 (80.0)	7 (20.0)	23 (65.7)	12 (34.3)	7 (20.0)	14 (40.0)	14 (40.0)
CR	2 (5.7)	1 (6.7)	1 (5.0)	0 (0.0)	2 (8.0)	2 (7.1)	0 (0.0)	1 (4.3)	1 (8.3)	0 (0.0)	1 (7.1)	1 (7.1)
PR	17 (48.6)	4 (26.7)	13 (65.0)	3 (30.0)	14 (56.0)	13 (46.4)	4 (57.1)	10 (43.5)	7 (58.3)	3 (42.9)	5 (35.7)	9 (64.3)
SD ≥ 24 w	3 (8.6)	2 (13.3)	2 (10.0)	1 (10.0)	3 (12.0)	3 (10.7)	1 (14.3)	3 (13.0)	1 (8.3)	2 (28.6)	1 (7.1)	1 (7.1)
SD < 24 w	8 (22.9)	5 (33.3)	2 (10.0)	4 (40.0)	3 (12.0)	5 (17.9)	2 (28.6)	4 (17.4)	3 (25.0)	1 (14.3)	5 (35.7)	1 (7.1)
PD	2 (5.7)	1 (6.7)	1 (5.0)	0 (0.0)	2 (8.0)	2 (7.1)	0 (0.0)	2 (8.7)	0 (0.0)	0 (0.0)	1 (7.1)	1 (7.1)
NE	3 (8.6)	2 (13.3)	1 (5.0)	2 (20.0)	1 (4.0)	3 (10.7)	0 (0.0)	3 (13.0)	0 (0.0)	1 (14.3)	1 (7.1)	1 (7.1)
ORR	19 (54.3)	5 (33.3)	14 (70.0)	3 (30.0)	16 (64.0)	15 (53.6)	4 (57.1)	11 (47.8)	8 (66.7)	3 (42.9)	6 (42.9)	10 (71.4)
CBR	22 (62.9)	7 (46.7)	16 (80.0)	4 (40.0)	19 (76.0)	18 (64.3)	5 (71.4)	14 (60.9)	9 (75.0)	5 (71.4)	7 (50.0)	11 (78.6)

*CR* complete response, *PR* partial response, *SD* stable disease, *PD* progressive disease, *NE* not evaluable, *ORR* overall response rate, *CBR* clinical benefit rate, *A/T* anthracycline- or taxane-based regimens, *W/O* without, *TN* triple negative, *yr* years, *Op* operation

**Fig. 2:**
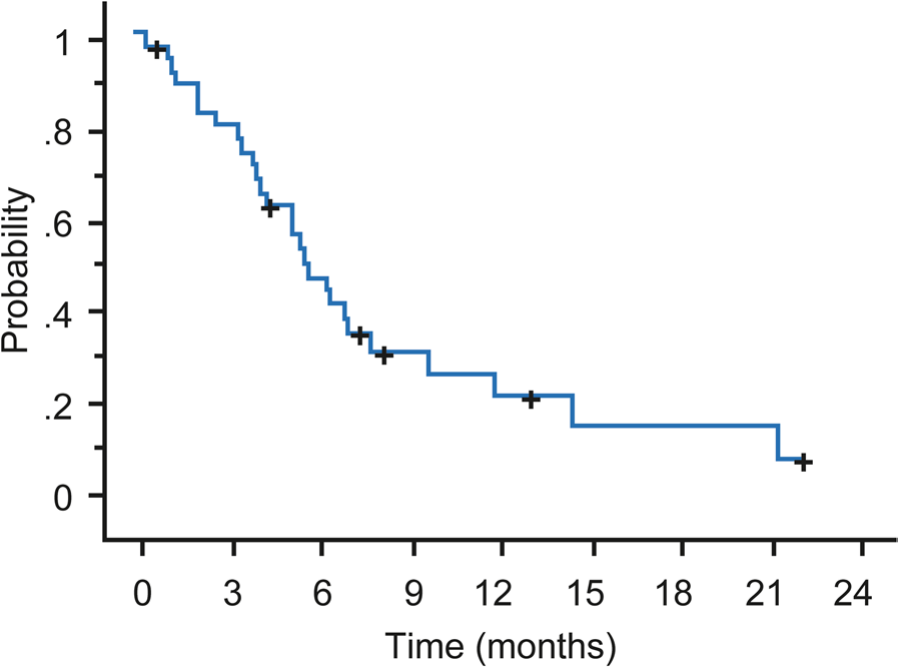
Kaplan–Meier plot of progression-free survival

**Fig. 3:**
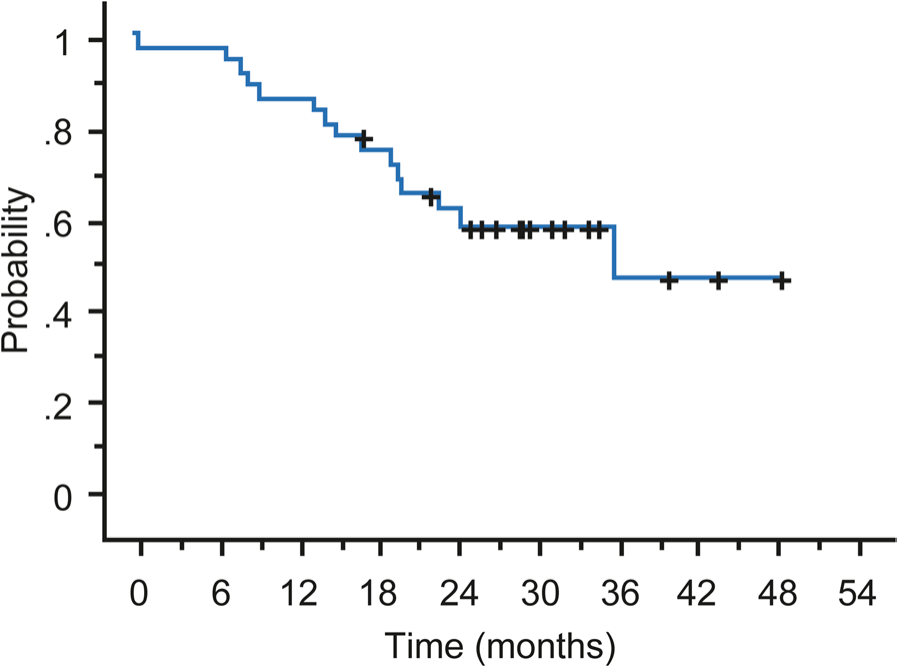
Kaplan–Meier plot of overall survival

The median PFS was 5.8 months (95 % CI 4.8–8.1) and median OS was 35.9 months. (Figs. 2, 3). The median TTF was 5.3 months (range 4.1–6.8), the median time to response was 1.4 months (range 1.2–3.7), and the median duration of response among patients who reached ORR was 4.6 months (range 0.6–22.1).

### Safety analysis

Observed adverse events are shown in Table 3. Hematological adverse events of any grade were reported in all of the patients. The most commonly reported grade 3 or 4 hematologic adverse event was neutropenia (22 patients; 62.9 %), followed by leucopenia (9 patients; 25.7 %). Febrile neutropenia was reported in two patients (5.7 %). The most commonly reported any grade non-hematological adverse event was alopecia (26 patients; 74.3 %), followed by fatigue (22 patients; 62.9 %), sensory neuropathy (21 patients; 60.0 %), and fever (17 patients; 48.6 %). Grade 3 or higher non-hematologic adverse events were reported in three patients (8.6 %); sensory neuropathy, mucositis, and skin rash in one patient (2.8 %) each. Five patients (14.3 %) discontinued eribulin therapy due to adverse events.

**Table 3:** Adverse events

Adverse events	N = 35	
	Any grade [n (%)]	Grade 3/4 [n (%)]
Hematological		
Neutropenia	34 (97.1)	22 (62.9)
Leucopenia	31 (88.6)	9 (25.7)
Anemia	18 (51.4)	0 (0)
Thrombocytopenia	18 (51.4)	0 (0)
Febrile neutropenia	2 (5.7)	2 (5.7)
Non-hematological		
AST	29 (82.9)	1 (2.9)
ALT	29 (82.9)	0 (0)
Alopecia	26 (74.3)	NA
Fatigue	22 (62.9)	0 (0)
Sensory neuropathy	21 (60.0)	1 (2.9)
Fever	17 (48.6)	0 (0)
Mucositis	13 (37.1)	1 (2.9)
Γ-GTP	13 (37.1)	2 (5.7)
Nausea	11 (31.4)	0 (0)
Taste disturbance	11 (31.4)	0 (0)
Anorexia	10 (28.6)	0 (0)
ALP	9 (25.7)	1 (2.9)
Skin rash	6 (17.1)	1 (2.9)
Vomiting	4 (11.4)	0 (0)
Constipation	4 (11.4)	0 (0)
Diarrhea	4 (11.4)	0 (0)
Arthralgia	4 (11.4)	0 (0)
Edema	4 (11.4)	0 (0)
Myalgia	3 (6.6)	0 (0)
Motor neuropathy	2 (5.7)	0 (0)

*NA* not available, *AST* aspartate aminotransferase, *ALT* alanine aminotransferase, *GTP* gamma-glutamyl transpeptidase, *ALP* gamma-glutamyl transpeptidase

Increases in laboratory values were reported as follows: aspartate aminotransferase (29 patients; 82.9 %), alanine aminotransferase (29 patients; 82.9 %), gamma-glutamyl transpeptidase (13 patients; 37.1 %), alkaline phosphatase (9 patients; 25.7 %), bilirubin (9 patients; 25.7 %), and albumin (9 patients; 25.7 %). Grade 3 events of increased aspartate aminotransferase, alkaline phosphatase, and albumin were reported in one patient each (2.9 %), and Grade 3 increase of gamma-glutamyl transpeptidase was reported in two patients (5.7 %). No Grade 3 increase of alanine aminotransferase or Grade 4 non-hematologic toxicity was reported. The majority of changes in laboratory values and vital signs were not clinically significant. There were no serious adverse events reported.

## Discussion

The current phase II study was to the first to investigate the efficacy and safety of eribulin as first-line chemotherapy for HER2-negative MBC in Japanese patients. The ORR and CBR were high, at 54.3 and 62.9 %, respectively. Interestingly, the ORR was higher in patients who had not received any neo/adjuvant chemotherapy, whose disease had luminal-like or triple negative features, or who had distant metastasis, than in those who had received neo/adjuvant chemotherapy. Hematological and non-hematological toxicities of any grade were reported in all of the patients; however, the majority of non-hematological adverse events were mild and tolerable. All reported adverse events were expected and no unexpected adverse events were reported.

The ORR and CBR (54.3 and 62.9 %, respectively) in this phase II trial were higher than those in a global phase II trial conducted by McIntyre et al. (28.6 and 51.8 %, respectively) (McIntyre et al. 2014), which might be due to differences in the proportion of patients who had received neo/adjuvant chemotherapy. In our trial, the ORR was higher in patients who had not received any neo/adjuvant chemotherapy than in those who had (70.0 vs. 33.3 %). Among patients who had received neo/adjuvant chemotherapy, patients who had not received anthracycline- or taxane-based regimens had higher ORR than those who had received anthracycline- or taxane-based neo/adjuvant chemotherapy (64.0 vs. 30.0 %). Our trial included a smaller proportion of patients who had received neo/adjuvant chemotherapy (43 %) than McIntyre’s trial (68 %). In addition, only 29 % of the patients included in our trial had received anthracycline- or taxane-based neo/adjuvant chemotherapy, while in McIntyre’s trial, 48 and 46 % of patients had received anthracycline- or taxane-based neo/adjuvant chemotherapy, respectively. Consequently, the lower proportion of patients in our trial who had received neo/adjuvant chemotherapy, especially anthracycline- or taxane-based neo/adjuvant chemotherapy, might have led to the higher ORR. Moreover, the ORRs were comparable to those with nanoparticle albumin-bound paclitaxel (42 %) and higher than those with paclitaxel (27 %) as first-line chemotherapy for MBC as shown in a phase III trial conducted outside Japan (Gradishar et al. 2005). The median time to response and duration of response (1.4 and 4.6 months, respectively) among patients who reached ORR in our trial were similar to those in McIntyre’s trial (1.4 and 5.8 months, respectively) (McIntyre et al. 2014).

The median PFS in this trial (5.8 months) was comparable to that reported in earlier clinical trials, including a study of first-line use of taxane (5.1 months) and anthracycline (7.2 months) for MBC (Piccart-Gebhart et al. 2008) and the phase II trial of eribulin conducted outside Japan (6.8 months) (McIntyre et al. 2014). Additionally, the median OS was 35.9 months in our trial, which seems to be comparable to taxane (37.2 months) and S-1 (35.0 months) for MBC in the Japanese population (Takashima et al. 2016). The survival benefit of eribulin has also been demonstrated in late-line therapy for MBC (Cortes et al. 2011; Kaufman et al. 2015; Twelves et al. 2014). One of the characteristics of eribulin is that it is associated with improvements in OS, but not PFS. This finding has also been noted with tumors other than breast cancer. A recent phase III trial of eribulin versus dacarbazine in patients with leiomyosarcoma and adipocytic sarcoma demonstrated that the median OS was significantly improved in patients treated with eribulin compared with those treated with dacarbazine, although median PFS was comparable between the patient groups (Schöffski et al. 2015). The survival benefit of eribulin might be due to improvement of the microenvironment of tumor cells, which was demonstrated by in vitro and in vivo preclinical studies (Funahashi et al. 2014; Yoshida et al. 2014; Terashima et al. 2014). Since one of the major goals of the therapy for MBC is to prolong survival, eribulin might be a suitable option to achieve this goal.

Overall, the safety of eribulin was acceptable, although five patients (14.3 %) discontinued therapy due to adverse events. The majority of non-hematological adverse events were mild in severity. Grade 3 or 4 sensory neuropathy, which might lead to discontinuation of eribulin therapy, was reported in only one patient. Among hematological adverse events, grade 3 or 4 neutropenia was reported in 62.9 % of patients; thus eribulin should be administered with caution and patients should be monitored closely for severe neutropenia. However, since febrile neutropenia was reported in only two patients, the tolerability of eribulin was considered to be acceptable. Notably, all reported adverse events were those that might be anticipated with this treatment and no new adverse events were reported in the first-line use of eribulin in this Japanese population. The proportion of patients who experience severe adverse events after initiation of eribulin is relatively low compared to that after initiation of other key drugs for MBC (Cortes et al. 2011; Kaufman et al. 2015; McIntyre et al. 2014). Thus, many patients treated with eribulin might not experience deterioration of their QoL. The current guidelines suggest using a single agent to optimize both treatment length and QoL for first-line therapy, except in the case of immediately life-threatening disease (Partridge et al. 2014; Cardoso et al. 2014). Japanese guidelines (The Japanese Breast Cancer Society 2015) also support this statement and the oral 5-fluorouracil derivative S-1 has become a recommended first-line treatment for MBC, along with anthracycline and taxane based on a recent clinical trial conducted in Japan—this trial demonstrated non-inferiority of S-1 in OS and TTF over taxane; S-1 also demonstrated less toxicity and better QoL profile compared to taxane (Takashima et al. 2016). In this context, eribulin might also be a recommended first-line treatment for MBC in the Japanese population, though further investigation is warranted.

Although the present trial offers meaningful data to evaluate efficacy and safety of first-line eribulin for treatment of HER2-negative MBC in Japanese patients, some caution is needed in the interpretation of the results. The present phase II trial was an exploratory study and conducted without any comparator. In addition, since the number of patients included in this trial was small (N = 35), caution is required for interpretation of OS data, due to lack of statistical power.

## Conclusion

In conclusion, the present phase II trial investigated the efficacy and safety of eribulin as first-line chemotherapy in Japanese women with HER2-negative MBC, and demonstrated that eribulin has antitumor activity comparable to that demonstrated by other key established cytotoxic agents. As eribulin has the potential to prolong survival in HER2-negative MBC patients, and has demonstrated acceptable safety and tolerability, it could be beneficial for such patients when used as a first-line therapy. Further research is necessary to confirm the results of the present phase II trial.

## Abbreviations

CBR: clinical benefit rate
CI: confidence interval
CR: complete response
HER2: human epidermal growth factor receptor 2
MBC: metastatic breast cancer
ORR: overall response rate
OS: overall survival
PFS: progression-free survival
PR: partial response
QoL: quality of life
SD: stable disease
TTF: time to treatment failure
